# Differential regulation of Type 1 and Type 2 mouse eosinophil activation by apoptotic cells

**DOI:** 10.3389/fimmu.2022.1041660

**Published:** 2022-10-31

**Authors:** Avishay Dolitzky, Inbal Hazut, Shmulik Avlas, Sharon Grisaru-Tal, Michal Itan, Ilan Zaffran, Francesca Levi-Schaffer, Motti Gerlic, Ariel Munitz

**Affiliations:** ^1^ Department of Clinical Microbiology and Immunology, Faculty of Medicine, Tel Aviv University, Tel Aviv, Israel; ^2^ Institute for Drug Research, School of Pharmacy, Faculty of Medicine, The Hebrew University of Jerusalem, Jerusalem, Israel

**Keywords:** eosinophils, allergy, inflammation, IL-4, IFN-gamma, apoptotic cells

## Abstract

Eosinophils are multifunctional, evolutionary conserved leukocytes that are involved in a plethora of responses ranging from regulation of tissue homeostasis, host defense and cancer. Although eosinophils have been studied mostly in the context of Type 2 inflammatory responses, it is now evident that they participate in Type 1 inflammatory responses and can respond to Type 1 cytokines such as IFN-γ. Notably, both Type 1- and Type 2 inflammatory environments are characterized by tissue damage and cell death. Collectively, this raises the possibility that eosinophils can interact with apoptotic cells, which can alter eosinophil activation in the inflammatory milieu. Herein, we demonstrate that eosinophils can bind and engulf apoptotic cells. We further show that exposure of eosinophils to apoptotic cells induces marked transcriptional changes in eosinophils, which polarize eosinophils towards an anti-inflammatory phenotype that is associated with wound healing and cell migration. Using an unbiased RNA sequencing approach, we demonstrate that apoptotic cells suppress the inflammatory responses of eosinophils that were activated with IFN-γ + *E. coli* (e.g., Type 1 eosinophils) and augment IL-4-induced eosinophil activation (e.g., Type 2 eosinophils). These data contribute to the growing understanding regarding the heterogeneity of eosinophil activation patterns and highlight apoptotic cells as potential regulators of eosinophil polarization.

## Introduction

Eosinophils are bone marrow-derived granulocytes, that are mainly present in mucosal surfaces such as the gastrointestinal and respiratory tract (1). Eosinophils can produce a plethora of immunoregulatory cytokines and are actively involved in the regulation of multiple immune responses (2). In response to diverse stimuli, eosinophils are recruited from the circulation into the inflammatory site, where they modulate immune responses through an array of mechanisms. For example, they can promote tissue damage or, conversely, encourage repair, which may eventually lead to tissue remodeling and fibrosis (3). Triggering of eosinophils by engagement of receptors for cytokines, immunoglobulins, and complement can lead to the generation of a wide range of inflammatory cytokines, lipid-derived mediators, and neuro-mediators (3). Thus, eosinophils can display direct or indirect effector functions by modulating multiple aspects of the immune response (1–3). Due to their ability to respond to, and secrete a multitude of mediators, eosinophils have pleotropic activities in numerous homeostatic processes (especially in mice) and display key roles in inflammatory responses that range from allergic diseases, host-defense and even cancer (4).

Eosinophils are considered terminally differentiated cells. Nonetheless, recent data demonstrate that exposure of eosinophils to distinct inflammatory microenvironments can induce differential transcriptional profiles in these cells (5–8). This could explain, at least in part their pleotropic activities in different disease contexts. For instance, exposure of eosinophils to cytokines such as IL-4, which is present in allergic diseases induces a unique transcriptome signature that is markedly different than the transcriptome signature that is induced by exposure of eosinophils to IFN-γ and/or innate immune stimulation (i.e., *E.* coli) (6). Based on these differential responses, we recently characterized the transcriptional profile of eosinophils and termed them Type-1 eosinophils (in response to IFN-γ with or without *E. coli* stimulation) and Type-2 eosinophils (in response to IL-4) (6). The presence of factors that are capable of modulating Type 1 and Type 2 eosinophil responses are largely unknown.

Cell death is a common feature of infected and damaged tissues in inflammatory sites (9). Engulfment of apoptotic cells by phagocytes (i.e., efferocytosis) (10) is a critical event in the resolution of inflammatory responses. The importance of efferocytosis in homeostasis is demonstrated by the finding that mice, which lack components that enable sensing, recognition and/or engulfment of dead cells, develop autoimmune diseases and/or chronic inflammation (11, 12). Indeed, efferocytosis can inhibit inflammatory signaling in macrophages and is associated with induction of tissue repair and wound healing. In agreement with this, the inflammatory response induced by lipopolysaccharide (LPS)-activated macrophages, is attenuated by their incubation with apoptotic cells (13). Conversely, optimal activation of macrophages with IL-4, which induces an anti-inflammatory phenotype in macrophages (e.g., M2 macrophages), requires the presence of apoptotic cells (14). Furthermore, apoptotic cells can induce the activation of STAT6, a key transcription factor in the IL-4/IL-13 signaling pathway (15).

Increased infiltration of eosinophils is observed in multiple diseases that are characterized by the presence of apoptotic cells. We have recently shown that eosinophils reside in the proximity of Caspase 3^+^ cells in the colons of mice with colorectal cancer (16). Furthermore, we have shown that eosinophils express various receptors mainly of the CD300 family (17–22), that may bind phosphatidylserine (PtdSer) (23), the most common ‘eat-me’ signal, which promotes the engulfment of apoptotic cells (24). Thus, eosinophils are potentially capable of detecting and responding to apoptotic cells. Whether apoptotic cells regulate transcriptional programs in eosinophils is largely unknown.

Herein, we demonstrate that eosinophils can bind and engulf to some extent apoptotic cells. We show that exposure of eosinophils to apoptotic cells induces marked transcriptional changes in eosinophils that are associated with wound healing and cell migration. Furthermore, using an unbiased RNA sequencing approach, we show that apoptotic cells suppress the inflammatory responses of Type 1 eosinophils and augment Type 2 eosinophil activation. These data contribute to the growing understanding regarding the heterogeneity of eosinophil activation patterns and highlight apoptotic cells as potential regulators of eosinophil polarization.

## Materials and methods

### Mice

Wild type (WT) C57BL/6 and BALB/c mice were obtained from Envigo, Israel. BALB/c *Cd300a^-/-^*, C57BL/6 *Cd300b^-/-^*, C57BL/6 *Cd300f^-/-^* were recently descried (19–22). NJ.1638* Il5^Tg^* mice (kindly provided by Dr. James L. Lee, Mayo Clinic, Phoenix, USA), were used for all studies using primary mouse cells. The mice were housed under specific pathogen-free conditions. All experiments were reviewed and approved by the Animal Health Care Committee of Tel Aviv University and were performed in accordance with the regulations and guidelines regarding the care and use of animals for experimental procedures.

### Eosinophil isolation

Mouse eosinophils were isolated from the peritoneal cavity of *Il5^Tg^* mice under sterile conditions (22). Peritoneal cavity was washed with 10mL of PBS. Thereafter, negative selection of eosinophils was performed using anti-Thy1.2 (11443D, Invitrogen) and anti-B220 (11331D, Invitrogen) Dynabead-conjugated antibodies according to the manufacturer’s instructions. Eosinophil purity was validated using flow cytometry; CD45-APC 07512-80-05 biogems; Siglec-F-PE Rat IgG2aκ, 552126/552128 BD Biosciences. Eosinophils were used when purity >97% and viability > 97%.

### Generation of bone marrow-derived eosinophils

Bone marrow-derived eosinophils (BMDEs) were generated as described (25). Briefly, bone marrow cells were collected from the femur and tibia bones by crushing and red blood cells will be lysed with ACK lysis buffer (Sigma). Low-density bone marrow progenitors were separated by gradient centrifugation (Histopaque 1083, Sigma) of 1700 RPM for 30 minutes. Low density bone marrow cells were seeded at 5 X 10^5^ cells/mL in 24 wells plate (BD Falcon) in media containing IMDM (Gibco) with 10% fetal bovine serum (HyClone), 1% penicillin-streptomycin (Biological industries), 2 mM glutamine (Gibco),50 μM β-mercaptoethanol (Sigma) and supplemented with 100 ng/mL stem-cell factor (SCF; PeproTech) and 100 ng/mL FLT3-Ligand (FLT3-L; PeproTech) from day 0 to day 4. On day 4, the media containing SCF, and FLT3-L replaced with media containing 10 ng/ml recombinant mouse interleukin-5 (rmIL-5; Peprotech) alone. Medium refreshing was done every 3 days from day 4 to 14 until eosinophil purity reached >85%. Purity was assessed by flow cytometry using CCR3 (R&D) and Siglec-F (BD Bioscience) as eosinophils markers.

### Generation of apoptotic cells

Wild-type mice (4- to 6-week-old) were euthanized and the thymus was collected, homogenized and thymocytes were incubated in an RPMI-1640 culture medium containing 0.1mM dexamethasone (Sigma). Following 6 hours of incubation, the cells were washed 3 times in the cell’s media by centrifugation.

### Annexin DAPI staining

After induction of apoptosis, thymocytes were washed once in PBS and suspended in Annexin binding buffer. Thereafter, the cells were stained with PE.Cy7-conjugated Annexin V (eBioscience) and DAPI as per the manufacturer’s instructions. After staining, cells were washed with Annexin binding buffer and suspended with PBS. Apoptotic cells were evaluated by flow cytometry and considered as AnnexinV^+^/DAPI^-^ cells.

### Efferocytosis and co culture assays

Eosinophils were labeled using CFSE - Cell Labeling Kit (Invitrogen, Carlsbad, CA) according to the manufacturer’s instructions. Apoptotic thymocytes were labeled using DiD (Invitrogen, Carlsbad, CA). Eosinophils and apoptotic cells were co-cultured (1:5 ratio, eosinophils:apoptotic cells) for 18 hours. Engulfment was determined by image-stream flow cytometry which captures single-cell images showing either single cells or engulfment. Live imaging was performed by snapping pictures every 5 min using IncuCyte^®^. All our stimulating conditions (including those of eosinophils without apoptotic cells) were conducted on eosinophil samples that were purified after the co culture by positive selection using anti-Siglec-F magnetic beads, and immediately resuspend in TRIzol™ Reagent (Invitrogen, Thermo Fisher). This procedure was conducted to ensure that apoptotic cells are not sequenced as well. Eosinophils from all the samples were then assessed by flow cytometry for their viability, which was >90% and purity >95%.

In other experiments, thymocytes were treated for 6 hours with Dexamethasone for the induction of apoptosis. Thereafter, the cells were washed and resuspended in PBS at a concentration of 10^6^/ml and labeled with 20nM pHrodo™ Red succinimidyl ester (SE) (Invitrogen, Thermo Fisher) by incubating them for 1 hour at 37^0^ C. The labeled cells were co-cultured with peritoneal eosinophils that were obtained from *Il5^Tg^* mice for 18 hours. Uptake was assessed by flow cytometry assessing cells that were positive for Siglec-F (as an eosinophils marker) and pHrodo™ Red, SE.

### Quantitative PCR analysis

RNA extracted using TRIzol Reagent (Invitrogen Life Technologies) and cDNA synthesis performed using the iScript™ cDNA Kit (Bio-Rad) according to the manufacturers’ instructions. PCR reactions were done using qPCR GreenMaster with ROX (Larova, Ornat). All primers are murine and were synthesized by Sigma-Aldrich Israel using the following primers: *Tim4:* Fwd (5’ 3’)- ATTCTCCCATCCACTTCACAG, Rev (3’-5’)- CACCATTAGCACAAATCCCAC (band size: 147bp); *Tim3*: Fwd-ACCCTGGCACTTATCATTGG, Rev- TTTTCCTCAGAGCGAATCCTG (band size: 149bp); *Cd300a: Fwd-*CAGGACCAACACTAGAGACAC, Rev-CAGGAGAGCTAACACAGACAAC (band size: 146bp); *Cd300b: Fwd-* AATGACACGGACACTTACTGG, Rev- CATGTCTGTACTGCCGTCC (band size: 150bp)*; Cd300c: Fwd-*AAGGTTGAGGTGTTCGTGG, Rev-CTTTCTGGTCACGCTGGG (band size: 150bp)*; Cd300d: Fwd-*CAGTTCTCTGCTCTACTCCTATTC, Rev-CTTGTAACCCTTCCAGTATGAGG (band size: 144bp)*; Cd300e: Fwd-*GTCTGCTCCTTCTCTGCTTC, Rev-GTCCTCGGCACCAGTATTTC (band size: 141bp)*; Cd300f: Fwd-*ACCACAGTAAAAGAGACCAGC, Rev-GAGATCCAGAAACCCATCACC (band size: 120bp)*; Cd300g: Fwd-*TCATTGTCTTTCCAGGGAGC, Rev-GGACAAGAGTATCAGGACTGG (band size: 149bp)*; MerTk: Fwd-*CCTGAGCCCGTCAATATCTTC, Rev-CGTCAGTCCTTTGTCATTGTG (band size: 144bp); *Axl: Fwd-*TGGGAGAAGGAGAATTTGGC, Rev-AGACAGCTTCACTCAGGAAATC (band size: 138bp). All qPCR reactions were conducted on a Bio-Rad CFX96 real-time PCR machine. Quantitation and normalization were relative to the housekeeping gene hypoxanthine-guanine phosphoribosyltransferase (*Hprt*).

### Flow cytometry

Flow cytometry was performed using a Gallios flow cytometer (Beckman-Coulter) to validate the expression level of selected surface markers on isolated eosinophils (3 X 105 cells in 200 μl). Staining was performed on ice for 30 minutes in HBSS supplemented with 1% BSA, 0.1% sodium azide. Data were analyzed using Kaluza analysis software on 10,000-50,000 acquired events. Surface molecule expression was calculated by defining the delta mean fluorescent intensity between the specific antibody stain and the isotype-matched control antibody.

The following antibodies were used to stain for PtdSer-recognizing cell surface receptors: Anti-mouse LMIR5/CD300b/CLM-7, Rat monoclonal clone 339003, MAB2580; Anti-mouse CD300c/d Rat IgG2bκ 148002, (Biolegend); Anti-mouse CD300LF/CLM-1 Goat polyclonal IgG Santa Cruz sc-161464; Anti-mouse TIM-4-PE Rat/IgG2a, kappa 12-5866 (eBioscience); Anti-mouse TIM-1 Rat/IgG2b, kappa 14-5861 (eBioscience).

### RNAseq

RNA was extracted using TRIzol™ Reagent (Invitrogen, Thermo Fisher) according to the manufacturer’s instructions. The RNA integrity number (RIN) was analyzed using Typestation (Agilent) and only samples of RIN>8 were used. RNA samples were prepared using the CEL-Seq2 protocol (26) with minor changes: instead of single-cells as input, 2 ng of purified RNA (obtained from 10^6 eosinophils), was used for library preparation. The CEL-Seq library was run on an Illumina NextSeq 550 apparatus according to manufacturer’s recommendation. The number of reads ranged from 3,093,819 to 10,621,072 per sample. The reads were mapped to the Mus musculus, GRCm38 genome (fasta:ftp://ftp.ensembl.org/pub/release97/fasta/mus_musculus/pep/Mus_musculus.GRCm38.pep.abinitio.fa.gzgtf ftp://%20ftp.ensembl.org/

pub/release97/gtf/mus_musculus/Mus_musculus.GRCm38.97.chr_patch_hapl_scaff.gtf.gz) using Tophat2 version 2.1.0 (27) with up to 2 mismatches allowed per read, the minimum and maximum intron sizes were set to 50 and 100,000, respectively, and an annotation file was provided to the mapper. The percentage of uniquely mapped reads ranged from 2,599,806 to 8,909,751 per sample. Only uniquely mapped reads were counted to genes, using ‘HTSeq-count’ package version 0.6.1 with ‘union’ mode (28). Normalization and differential expression analyses were conducted using DESeq2 R package version 1.10.0 (29). Sample preparation, sequencing, quality control, and normalization were conducted by the Technion Genome Center, Life Science and Engineering Interdisciplinary Research Center, Technion, Haifa, Israel.

### Bioinformatics analysis

RNA-Seq data from the experiment was trimmed using fastp 0.20.1 (30) and aligned using STAR 2.7.2a (31). Normalization and differential expression analyses were conducted using DESeq2 R package version 1.32.0. Genes were regarded as statistically significant and differentially expressed if they presented false discovery rate (FDR) lower than 0.05 and changed their expression by a factor of 1.5 or more (6). P values were adjusted with FDR multiple comparison correction. Gene ontology annotations were obtained from Ensembl and pathway graphs were obtained from KEGG. In several analyses, datasets were retrieved from public domains and therefore not all genes were identified. In such cases NA represents non-applicable. All datasets presented in this study are available online in accession accession numbers: GSE189213 and GSE216110.

### Enzyme-linked immunosorbent assay

Cytokines were measured by enzyme-linked immunosorbent assay (ELISA) according to the manufacturer’s instructions kit: IL-6, TNF-α, and CCL17 (R&D Systems, Minneapolis, MN).

### Statistical analysis

*P* values of data sets were adjusted with false discovery rate (FDR) using multiple comparison correction (32). FDR lower than 0.05 and fold-change by a factor of 1.5 or more were analyzed. In *in vitro* experiments, one-way analysis of variance (ANOVA), unpaired two-tailed Student’s *t* test with 95% confidence interval were used. All statistical tests were performed with GraphPad Prism V8 software. Experiments are from n=3 biological replicates. Data are shown as mean ± SEM. *-p < 0.05; **-p < 0.01; ***-p* < 0.001*.

## Results

### Eosinophils bind and engulf apoptotic cells

**Figure 1:**
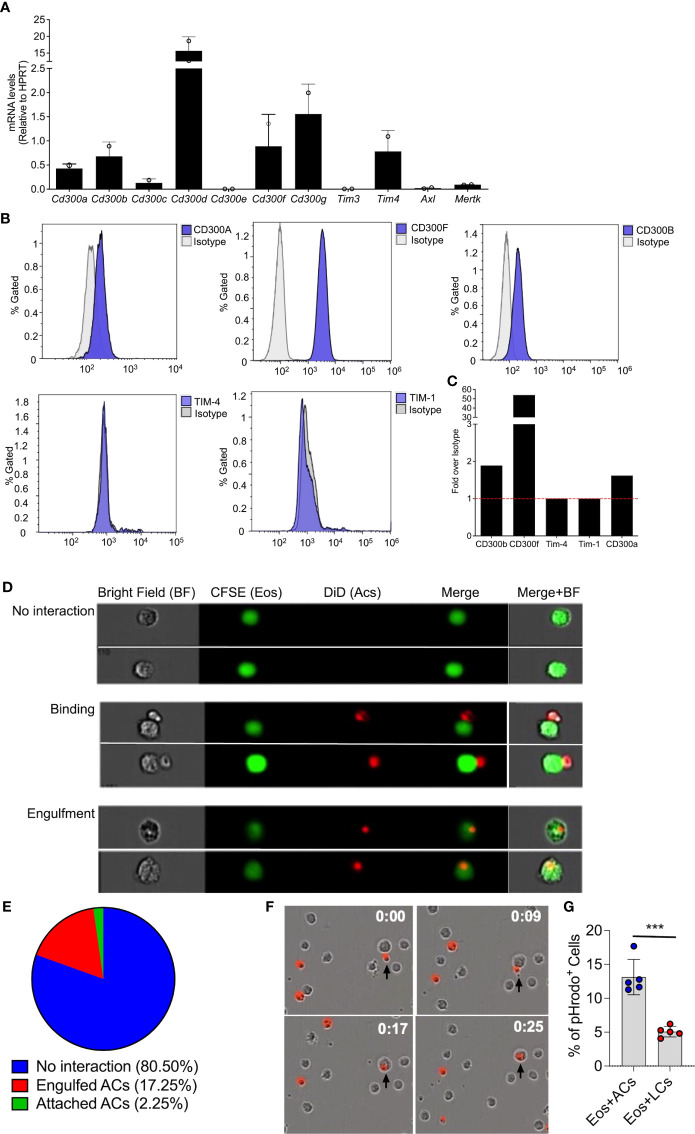
Eosinophils bind and engulf apoptotic cells. mRNA expression of various phosphatidylserine binding receptors was determined in eosinophils by quantitative PCR analysis and normalized to the expression of the house keeping gene hypoxanthine-guanine phosphoribosyltransferase (*Hprt*) **(A)** Protein expression of CD300a, CD300b, CD300f, TIM-1 and TIM-4 was determined by flow cytometry **(B)** and presented as fold expression over isotype control **(C)** Representative photomicrographs **(D)** obtained from ImageStream analysis of CFSE-labeled eosinophils (Eos, green) interacting with DiD-labeled apoptotic cells (red) **(D)**. Quantitative analysis of eosinophil-apoptotic cell interactions is presented **(E)** Snap-shot images from time lapse microscopic analysis of eosinophils (unstained) engulfing DiD-labeled apoptotic cells (red; time-0, 9, 17 and 25 min) **(F)** apoptotic cells (ACs) and live cells (LCs) were labeled with pHrodo™ SE Red and co cultured with eosinophils. The percentage of pHrodo™ SE Red positive cells is shown **(G)** Data are from n=3 **(A, E)** or representative images from n=3 independent experiments. In **(G)**, n=5. ***- p< 0.001.

Extracellularly exposed PtdSer is recognized by multiple cell surface receptors and bridging molecules, such as CD300-family members, T-cell immunoglobulin mucin (Tim) receptors, brain-specific angiogenesis inhibitor 1 (BAI1), and or Tryo3-Axl-Mer (TAM) receptors (23, 33–35). Thus, we were first interested to determine the expression pattern of PtdSer-recognizing receptors in eosinophils. To this end, RNA was extracted from peritoneal eosinophils and mRNA expression of CD300-family members, TIM-3, TIM-4, AXL and MerTK were assessed by quantitative PCR. Eosinophils expressed mRNA for multiple PtdSer-recognizing receptors including *Cd300a*, *Cd300b*, *Cd300d*, *Cd300f*, *Cd300g*, *Tim4*, and to and to lesser extent *Cd300c* and *Mertk* (**Figure 1A**). Next, we aimed to confirm protein expression of the aforementioned receptors. Flow cytometric analysis revealed that eosinophils express detectable levels of CD300a, CD300b, CD300f but do not express TIM-4 and TIM-1(**Figures 1B, C**).

Expression of PtdSer-binding receptors on eosinophils suggest that they may interact with apoptotic cells. Thus, we determined whether eosinophils bind and/or engulf apoptotic cells. Peritoneal eosinophils were isolated from *Il5^Tg^* mice and labeled with CFSE. Subsequently, the eosinophils (marked in green color) were co-cultured with DiD-labeled apoptotic cells (marked in red) and eosinophil-apoptotic cell interactions was measured by image stream (**Figure 1D**), which enables to capture single-cell images of interacting cells. This analysis revealed eosinophils in three different states. Namely, eosinophils that have no interaction with apoptotic cells (**Figure 1D**- upper panel), Eosinophils that are attached to apoptotic cells (**Figure 1D**- middle panel) and eosinophils that engulfed apoptotic cells (**Figure 1D**- lower panel). Quantitative analysis showed that approximately 20% of the eosinophils were either attached to apoptotic cells (2.25%) or engulfed apoptotic cells (17.25%) (**Figure 1E**). The ability of eosinophils to bind and engulf apoptotic cells was further established using real-time, live-cell imaging analysis where upon introduction of DiD-labeled apoptotic cells (**Figure 1F**, time 0:00), eosinophils were capable of binding them (**Figure 1F**, time 0:09) and rapidly engulfing them (**Figure 1F**, time 0:17 and 0:25).

To further confirm the ability of eosinophils to uptake dead cells, the uptake of pHrodo-labeled *thymocytes* was measured *in vitro* by flow cytometry. This method uses a unique pHrodo dye that fluoresces in response to an acidic environment that is found in the phagosome (36). Indeed, ~13% of the eosinophils, which were co-cultured with apoptotic cells were postive for pHrodod (**Figure 1G**).

### Apoptotic cells induce transcriptional changes in eosinophils

**Figure 2:**
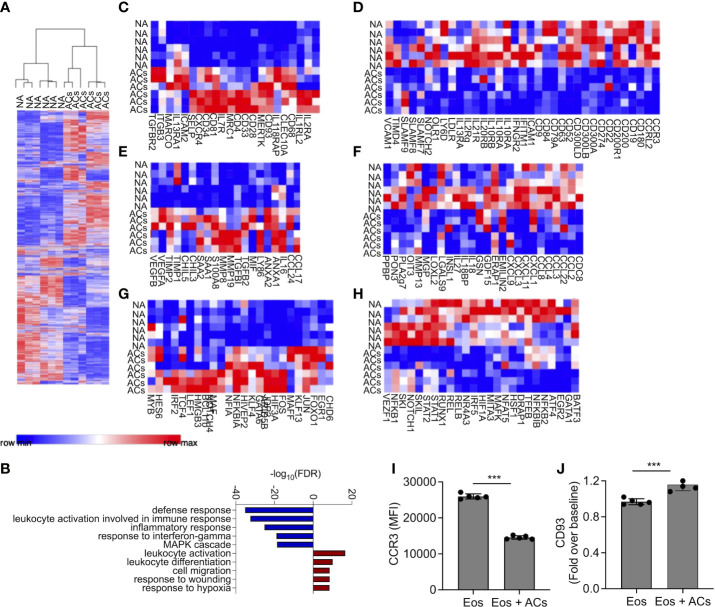
Apoptotic cells induce transcriptional changes in eosinophils. Heat-plot analysis of statistically significant (≥≤ +/-1.5-fold, adj. p value ≤ 0.05) differentially expressed transcripts of eosinophils that were co-cultured with apoptotic cells (ACs) in comparison to non activated eosinophils (NA) **(A)** In **(B)**, Gene ontology (GO) analysis based on biological processes (BP) using the statistically significant differently express transcripts that were induced by apoptotic cells is shown. Heat plot analysis of apoptotic cell-induced up and downregulated cell surface receptors **(C, D)**, secreted factors **(E, F)**, and transcription factors **(G, H)** of eosinophils following incubation with apoptotic cells. Expression of CCR3 **(I)** and CD93 **(J)** was determined by flow cytometry on the surface of eosinophils, which were incubated in the presence of apoptotic cells. Each lane indicates eosinophils that were obtained from a different mouse. ***- p< 0.001.

Following the observation that eosinophils can interact with apoptotic cells, we were interested to determine whether interaction of eosinophils with apoptotic cells will alter their transcriptional profile. To this end, mouse eosinophils were incubated with apoptotic cells and subjected to RNA sequencing. Pairwise comparison analysis revealed that eosinophils that were co cultured with apoptotic cells upregulated 1,795 transcripts and downregulated 1,678 transcripts, (adjusted p-value < 0.05 and fold change of >< ± 1.5, **Figure 2A** and **Table S1**). The list of up- and down-regulated transcripts were further analyzed using gene ontology (GO) based on biological processes. This analysis identified that upon interaction with apoptotic cells, eosinophil upregulate pathways that are related to global “leukocyte activation” including “response to wound healing” and “cell migration” (**Figure 2B**). In contrast, GO analysis of the biological processes that were dictated by the downregulated transcripts indicated that following interaction with apoptotic cells, eosinophils downregulated pathways related to “defense response”, “response to IFN-γ”, and “inflammatory response” (**Figure 2B**). These data are consistent with the anti-inflammatory effects of apoptotic cells on additional myeloid cell types (10, 13). To further characterize the transcriptional changes induced in eosinophils by apoptotic cells we analyzed the expression of cell surface receptors (**Figures 2C, D**, **Supplementary Tables 2** **–** **3**), Secreted factors (**Figures 2E, F**, **Supplementary Tables 4** **–** **5**), and transcription factors (**Figures 2G, H**, **Supplementary Tables 6** **–** **7**).

#### Cell surface receptors

Following incubation with apoptotic cells eosinophils upregulated the expression of *Cd34*, which we previously identified as a Type-2 activated eosinophil marker (6) (**Figure 2C**). In addition, the expression of several cytokine and chemokine receptors including *Il2ra, Il7r Il18rap, Il1rl2, Il13ra1, Tgfbr2* and *Cxcr4*, was increased. Apoptotic cells also increased the expression of adhesion molecules such as *Selp* (P-selectin), *Itgb3*, and *Icam2*, (**Figure 2C**). Interestingly, apoptotic cells markedly downregulated the expression of CD300 family members including *Cd300a, Cd300lb* and *Cd300ld* (**Figure 2D**). Furthermore, multiple SLAM-family receptors such as *Slamf7, Slamf8 and Slamf9* were decreased (**Figure 2D**). *Ccr3*, the main receptor for eotaxin chemokines (37), which have key roles in eosinophil biology was downregulated as well (38). Finally, and consistent with the overall anti-inflammatory effects that are associated with the interactions of immune cells with apoptotic cells, the expression of multiple IFN-γ-related transcripts including *Ifitm1*, *Ifngr2*, and the recently described Type1-activated eosinophil marker *Cd274* (PD-L1) were decreased.

#### Secreted factors

Incubation of eosinophils with apoptotic cells induced the expression of soluble factors that are associated with Type-2 immune responses and tissue repair. For example, the IL-4/IL-13-associated factors *Ccl17*, *Ccl24*, *Chil3*, and *Chil5 (* 39 *)* were upregulated (**Figure 2E**, **Table S4**). *S100a8*, which was previously shown to be highly upregulated by eosinophils during colonic repair (8), was also upregulated. In addition, the pro-fibrotic factors/anti-inflammatory factors *Tgfbi*, and *Tgfb2 (* 40 *)*, were also increased following interaction with apoptotic cells. Interestingly, vascular endothelial growth factor (*Vegf*)a/b, which induces angiogenesis (41), and *Anxa1/2*, a regulator of innate immune responses, were also increased (42). Apoptotic cells decreased the expression of numerous pro-inflammatory secreted factors (**Figure 2F**, **Table S5**). These include multiple chemokines (e.g., *Cxcl1, Cxcl3 Cxcl9, Cxcl11, Ccl2, Ccl3, Ccl4 Ccl8, and Ccl22*) and cytokines (e.g., *Il18* and *Il27*) (**Figure 2F**).

#### Transcription factors

Transcription factor analysis revealed that apoptotic cells increased the expression of *Gata6* and *Maf (* 43, 44 *)*, which are associated with Th2 activation. The expression of *Klf4*, which is associated with M2 macrophage polarization was increased as well, and several wnt- and *Notch-*family associated transcription factors such as *Lef1* and *Notch4* were increased (**Figure 2G**). Consistently, apoptotic cells downregulated the expression of several pro-inflammatory transcription factors (**Figure 2H**). These include the signal transducer and activator of transcription (STAT) family members, *Stat1* and *Stat2*. Furthermore, *Nfkb1* and *Nfkb2* were also downregulated (**Table S7**).

To confirm our RNA sequencing data, we chose to examine protein expression of CD93 and CCR3 which were suggested to be up- and downregulated by apoptotic cells, respectively (**Table S2** **–** **3**). Incubation of eosinophils with apoptotic cells decreased cell surface expression of CCR3 and increased CD93 expression (**Figures 2I, J**). Collectively, these data suggest that apoptotic cells suppress pro-inflammatory genes activation on eosinophils and may augment their responses in settings of tissue repair and remodeling.

### Apoptotic cells suppress proinflammatory responses in Type 1-activated eosinophils

**Figure 3:**
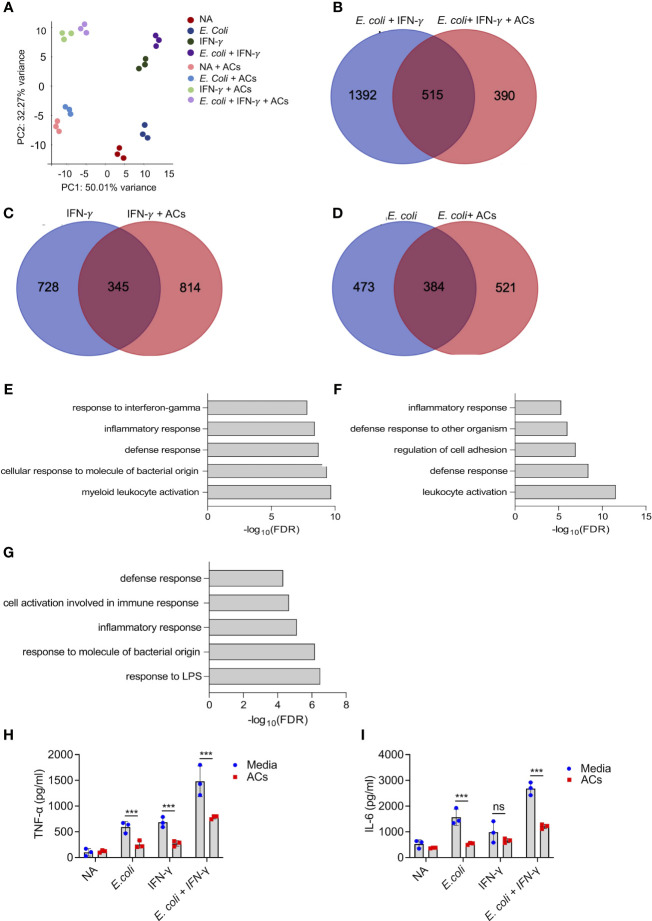
Apoptotic cells suppress proinflammatory responses in Type 1-activated eosinophils. Principal components analysis (PCA) of differentially expressed transcripts following incubation of non activated eosinophils (NA) or eosinophils with *E coli*, IFN-γ, *E coli*+ IFN-γ, in the presence or absence of apoptotic cells (ACs, A). Venn-plot representation compering the transcriptome signatures of transcripts that were upregulated in eosinophils by *E coli* + IFN-γ, *E coli*, or IFN-γ, with the transcripts that downregulated when *E coli* + IFN-γ, *E coli*, or IFN-γ stimulated eosinophils in the presence of apoptotic cells **(B-D)**. Gene ontology (GO) analysis based on biological processes (BP) using the statistically significant transcripts inhibited by apoptotic cells following activation with *E coli* + IFN-γ, *E coli*, or IFN-γ **(E-G)**. Purified eosinophils were stimulated with *E coli* + IFN-γ, *E coli*, or IFN-γ in the presence or absence of apoptotic cells (ACs). Subsequently, the secretion of TNF-α **(H)** and IL-6 **(I)** were determined by ELISA. In **(A)**, each dot represents a different sample (n=3). In **(H, I)** data are representative of n=3 different experiments conducted in triplicates; ns- nonsignificant, ***p<0.001.

Next, we aimed to determine whether apoptotic cells could suppress proinflammatory responses in eosinophils following stimulation with IFN-γ and/or *E. coli*. To this end, eosinophils were stimulated with IFN-γ, *E. coli*, and IFN-γ followed by *E.* coli to elicit Type 1-polarized eosinophils (6). We specifically classify Type 1 eosinophils as cells that are exposed to IFN-γ in the presence of bacterial/innate stimuli since this condition has been widely used to characterize M1 macrophages (45). Thereafter, the cells were subjected to RNA sequencing. Principal component analysis (PCA) analysis, which represents the relationship between biological replicates, revealed that the major factor, which was responsible for the differences between the samples was the presence of apoptotic cells (**Figure 3A**). This is demonstrated by the PC1 axis that segregated between the samples according to the presence or absence of apoptotic cells and represents 50% of the variance between all groups (**Figure 3A**). Using Venn plot analysis, we compared the effects of apoptotic cells on transcripts that were upregulated in eosinophils following activation with IFN-γ with or without *E. coli*. (**Figures 3B–D**). Apoptotic cells suppressed the expression of 515 transcripts, which were upregulated by eosinophils that were stimulated with *E. coli* and IFN-γ (**Figure 3B**, **Table S8**). Apoptotic cells inhibited the expression of several surface markers that are associated with an inflammatory phenotype of eosinophils (**Table S8**). For instance, *Cd274*, a key Type-1 activated eosinophil marker (6, 32), was decreased by ~2.4-fold following interaction with apoptotic cells. Moreover, the expression levels of *Cd83* and *Cd86* were downregulated by ~3.8 and ~1.5 respectively. In addition, the expression of the antiviral transmembrane proteins *Ifitm1* and *Ifitm2* were also decreased by apoptotic cells, (~2.7 and ~1.5-fold, respectively). Notably, apoptotic cells inhibited the expression of proinflammatory soluble mediators including *Il1a*, *Il1b*, *Il6*, *Il12b*, *Cxcl1*, *Cxcl2*, and *Tnfa*. Furthermore, apoptotic cells downregulated the expression levels of several hallmark inflammatory transcription factors such as *Nfkb2*, *Mapk*, *Mapkapk2*, *Ppard*, and Lipopolysaccharide-Induced TNF-α (*Litaf*) (**Table S8**). The anti-inflammatory effects of apoptotic cells on eosinophils were also observed towards stimulation of eosinophils with IFN-γ or *E. coli* as single activation agents. Out of 1,073 transcripts that were upregulated by IFN-γ, apoptotic cells downregulated the expression of 345 (32%, **Figure 3C**, **Table S8**) and out of 857 transcripts that were upregulated by *E. coli*, apoptotic cells decreased the expression of 384 (45%) of total (**Figure 3D**, **Table S8**). Down regulated transcripts in response to IFN-γ stimulation included surface markers (e.g., *Cd36*, *Ldlr*, *Olr1*, *Ly6g*, and *Tlr6*), proinflammatory chemokines/cytokines (e.g., *Cxcl2*, *Cxcl3*, *Il1a*, *Il1b*, *Mif*, and *Il6*), and transcription factors (e.g., *Mapk7*, *Nfkb2*, *Stat1*, *Stat5b*, I*rf1*, and *Nlrc5*). Similarly, down regulated transcripts in response to *E. coli* stimulation included surface markers such as *Cd274* and *Pdcd1lg2* (PD-L2), the pro-inflammatory receptors *Cd80* and *Ldlr* and the LPS receptor *Tlr4* were also downregulated. Interestingly, *Csf2rb* (The common beta chain for IL-3, IL-5 and GM-CSF), was also decreased by 1.69-fold. Moreover, proinflammatory secreted factors (e.g., *Ccl2*, *Ccl3*, *Cxcl1*, *Cxcl2*, *Cxcl3*, I*l1a*, *Il6*, and *Tnfa*), were downregulated.

To further characterize the effects of apoptotic cells on the suppression inflammatory pathways in Type-1 activated eosinophils, a bioinformatics gene ontology pathway analysis was conducted. The pathways, which were suppressed by apoptotic cells were associated with “response to IFN-γ”, “Inflammatory response”, “response to LPS”, and “defense response” (**Figures 3E–G**). To functionally validate the ability of apoptotic cells to inhibit Type 1 eosinophil responses, eosinophils were stimulated with IFN-γ, *E. coli* and IFN-γ with *E. coli.* Stimulation of eosinophils with IFN-γ, *E. coli*, and *E. coli* + IFN-γ resulted in increased the secretion of TNF-α and IL-6, Incubation of eosinophils with apoptotic cells markedly suppressed IFN-γ-, *E. coli-* and IFN-γ+*E.coli*-induced secretion of TNF-α and IL-6 (**Figures 3H, I**).

### Apoptotic cells augment Type 2 eosinophil activation

**Figure 4:**
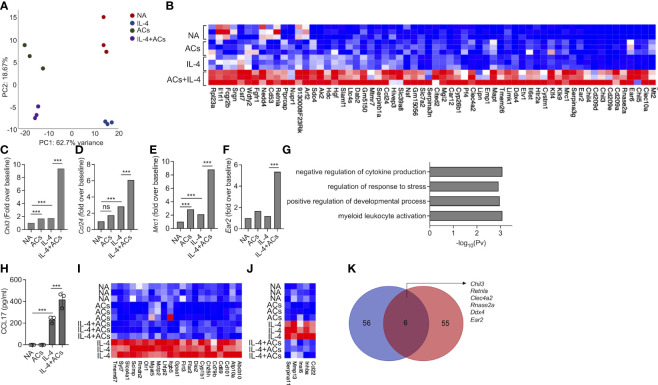
Apoptotic cells augment Type 2 eosinophil activation. Principal components analysis (PCA) of differentially expressed transcripts following incubation of non activated eosinophils (NA) or eosinophils stimulated with IL-4, apoptotic cells (ACs) or IL-4 in the presence of apoptotic cells **(A)**. Heat-map representation of the set of transcripts (62 transcripts) that were augmented by ACs following stimulation with IL-4 compared with apoptotic cells alone and IL-4 alone **(B)**. mRNA levels, of Type-2 eosinophil-associated transcripts, as identified by RNAseq **(C-F)**. Gene ontology (GO) analysis based on biological processes (BP) using the statistically significant differently expressed transcripts following which were increased in eosinophils following their incubation with IL-4 and ACs in in comparison with IL-4 **(G)**. Purified eosinophils were either not activated (NA) or stimulated with IL-4, ACs or the combination of IL-4 + ACs. Thereafter, the secretion of CCL17 was determined by ELISA **(H)**. Heat-map representation of the set of transcripts that were decreased by ACs following stimulation with IL-4 compared with IL-4 alone **(I, J)**. Venn-plot representation comparing the set of transcripts, which were augmented by ACs in the presence of IL-4 in eosinophils (Eos) and macrophages (Mac) **(K)**. In **(A)**, each dot represents a different sample (n=3). In **(H)** data are representative of n=3 different experiments conducted in triplicates; ns- nonsignificant, ***p<0.001.

Recently, apoptotic cells were shown to enhance macrophage responses towards IL-4. Thus, we were interested to determine whether apoptotic cells will increase IL-4-induced responses in eosinophils as well. To this end, eosinophils were stimulated with IL-4 in the presence or absence of apoptotic cells. Thereafter, RNA was extracted and subjected to RNA sequencing. PCA plot of the top 500 variable genes, revealed that the factor, which was accounted for the major variance between the samples (62.7%), was explained by the presence of apoptotic cells (**Figure 4A**). PC2 that represents the differences induced by IL-4, accounted for only 18% of the variance (**Figure 4A**). The PCA plot further demonstrated that the groups, which displayed the largest difference were unstimulated eosinophils and eosinophils stimulated with IL-4 in the presence of apoptotic cells (**Figure 4A**). Our analyses revealed that 62 transcripts were significantly upregulated by the combination of IL-4 and apoptotic cells compared to IL-4 or apoptotic cells alone (**Figure 4B**, **Table S9**). Among these transcripts, several were associated with tissue remodeling and repair. For example, IL-4 increased the expression of *Chil3* by 1.7-fold compared to unstimulated cells. The presence of apoptotic cells induced *Chil3* expression to 9.3-fold (**Figure 4C**, **Table S9**). Similarly, the expression of *Chil4* and *Chil5* were also augmented from a fold increase of 4.1 and 1.4 with IL-4 alone to ~20.5 and ~17.5 with IL-4 in the presence of apoptotic cells. Combination of apoptotic cells and IL-4 also increased the expression of hallmark eosinophil-associated transcripts (e.g., *Ccl24* and *Ear2*) and several markers of alternatively activated macrophages such as *Cd209*, *Retnla*, *Klf4, Serpina3, Car12* and *Mrc1 (* 46, 47)(**Figures 4D–F**). Gene ontology (GO) analysis based on biological process demonstrated that stimulation of eosinophils with apoptotic cells and IL-4 enriched pathways that are associated with “negative regulation of cytokine production”, “regulation of response to stress” and “regulation of developmental process” (**Figure 4G**). To functionally validate our RNA sequencing data, secretion of CCL17, an IL-4-induced chemokine, was determined following stimulation of eosinophils with IL-4 or IL-4 and apoptotic cells. As expected, IL-4 induced the secretion of CCL17 (**Figure 4H**). Addition of apoptotic cells to IL-4 enhanced CCL17 secretion (**Figure 4H**).

Stimulation of eosinophils with IL-4 in the presence of apoptotic cells resulted also in decreased expression of 69 transcripts (**Figure 4I**, **Table S10**). Among these, the expression of *Cd69*, an eosinophil activation marker and expression of *Cd101*, a recently described Type 2 eosinophil marker and *Ccl22*, an IL-4-induced chemokine were decreased (**Figures 4I, J**). GO analysis of the downregulated transcript signature resulted in no enrichment of a specific biological pathway (data not shown).

Next, we examined whether the 62 transcripts, which were amplified in eosinophils by stimulation by IL-4 in the presence of apoptotic cells were similar to that, which was previously shown for macrophages (14). IL-4 stimulation in the presence of apoptotic cells induced a minor overlap in transcript identity between eosinophils and macrophages (**Figure 4K**). Nonetheless, the shared transcripts (i.e., *Chil3*, *Retnla*, *Clec4a2*, *Rnase2a*, *Ddx4*, and *Ear2*) (**Figure 4K**, **Table S10**) were previously associated with tissue repair and remodeling activities tissue repair-related genes. Taken together, these findings suggest that apoptotic cells enhance eosinophil activities in Type-2 inflammatory settings.

### Eosinophil recognition of apoptotic cells is independent of CD300b and CD300f

**Figure 5:**
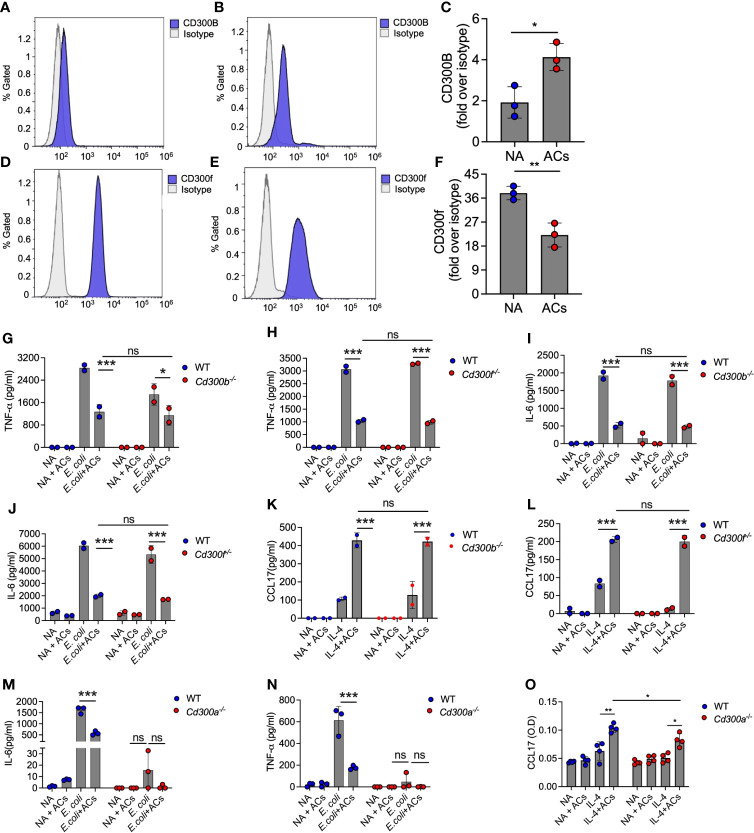
Eosinophil recognition of apoptotic cells is independent of CD300b and CD300f. Surface expression and quantitative analysis of CD300b **(A–C)** and CD300f **(D–F)** following incubation of eosinophils with apoptotic cells (ACs) was determined by flow cytometry. Purified eosinophils from wild type (WT), *Cd300b^-/-^* **(G, I, K)**, *Cd300f^-/-^* **(H, J, L)**, or *Cd300a^-/-^* **(M–O)** were obtained and stimulated with *E. coli* **(G-J, M, N)** or IL-4 **(K, L, O)** in the presence or absence of apoptotic cells (ACs). Thereafter, the secretion of TNF-α **(G, H, N)**, IL-6 **(I, J, M)** and CCL17 **(K, L, O)** were determined by ELISA. In **(A)**, each dot represents a different sample (n=3). In **(A, B, D, E)** data are representative of n=3 different experiments, which are summarized in **(C–F)**. In all other experiments, data are representative of n=3 different experiments conducted in triplicates; ns- nonsignificant, *p<0.05, **p<0.01, ***p<0.001.

Eosinophils express various CD300 receptor family members that could potentially interact with apoptotic cells *via* recognition of PtdSer with specifically high levels of CD300f [**Figures 1B, C** and (18–22, 48)]. Hence, we examined whether apoptotic cells suppress Type 1 eosinophil activation or augment Type 2 eosinophil activation *via* these CD300-family receptors. Our RNA sequencing data suggested that apoptotic cells decreased the expression of CD300-family members. Interestingly, exposure of eosinophils to apoptotic cells markedly increased cell surface expression of CD300b but decreased CD300f expression (**Figures 5A–F**).

Given the relative high expression level of CD300f in eosinophils and the finding that CD300b expression was increased following interaction with apoptotic cells, we hypothesized that CD300b and CD300f will mediate apoptotic cell recognition in eosinophils. Despite high mRNA expression of CD300d in eosinophils we did not functionally examine its involvement in eosinophil-apoptotic cell interactions since it was previously shown to be retained intracellularly, and we could not detect any surface expression of CD300d in eosinophils (data not shown). To examine this, eosinophils from WT, *Cd300b^-/-^* and *Cd300f^-/-^* mice were obtained and stimulated with *E. coli* or IL-4. Apoptotic cells were capable of suppressing *E. coli*-induced secretion of IL-6 and TNF-α in *CD300b^-/-^* and *Cd300f^-/-^* eosinophils to a similar extent as in WT BMDEs (**Figures 5G–J**). Simulation of *Cd300b^-/-^* BMDEs with IL-4 in the presence of apoptotic cells induced similar levels of CCL17 secretion as in WT BMDEs stimulated with IL-4 and apoptotic cells (**Figure 5K**). Consistent with our previous data, *Cd300f^-/-^* BMDEs did not respond to IL-4 stimulation (20). Nonetheless, the presence of apoptotic cells restored the responses of *Cd300f^-/-^* BMDEs to IL-4 to a similar extent as WT eosinophils (**Figure 5L**). These data demonstrate that CD300b and CD300f do not mediate apoptotic cell-driven responses in eosinophils.

Surprisingly, *Cd300a^-/-^* BMDEs did not respond to *E. coli*-induced stimulation, and we could not detect TNF-α or IL-6 in the culture supernatants (**Figures 5M, N**). Similarly, although IL-4-stimulated *Cd300a^-/-^* BMDEs displayed slightly reduced levels of CCL17 secretion in comparison with IL-4-stimulated WT BMDEs, addition of apoptotic cells augmented CCL17 secretion in *Cd300a^-/-^* and WT BMDEs to a similar extent (**Figure 5O**).

## Discussion

Eosinophils are white blood cells that are traditionally associated with allergic and parasitic diseases. Nonetheless, accumulating data suggest key roles for eosinophils in homeostasis and host defense. Since eosinophils are considered terminally differentiated cells, much knowledge has been gained regarding the signals which mediate eosinophil survival and prevent apoptosis. Much less is known regarding the ability of eosinophils to interact with apoptotic cells in the microenvironment and how these interactions can shape eosinophil activities. This is of specific interest since clearance of apoptotic cells and engulfment of apoptotic cells by phagocytes is a key event in the resolution of inflammatory responses (10, 13). The importance of efferocytosis in homeostasis is further demonstrated by the finding that mice, which lack components that enable sensing, recognition and/or engulfment of dead cells, develop autoimmune diseases and/or chronic inflammation (49). Like additional immune cells, eosinophils are constantly exposed to apoptotic cells that are present in their microenvironment (1). Thus, we aimed to characterize the effects of apoptotic cells on eosinophil activities. We demonstrate that eosinophils can physically bind and engulf apoptotic cells. Furthermore, using an unbiased global RNA sequencing approach we show that interaction of eosinophils with apoptotic cells renders them anti-inflammatory. Stimulation of eosinophils with Type 1 polarizing agents (i.e., IFN-γ, *E. coli* and combinations of thereof) (6) in the presence of apoptotic cells markedly suppressed their proinflammatory transcriptome signature and ability to secrete inflammatory cytokines. Furthermore, apoptotic cells augment eosinophil responses towards IL-4, which induces a transcriptome signature that is associated with tissue repair and remodeling. Taken together our data demonstrate that apoptotic cells shape the transcriptome landscape of eosinophils by polarizing eosinophils into an anti-inflammatory phenotype.

Using high resolution and live-imaging techniques, we show that eosinophils can bind and engulf to some extent apoptotic cells. The interaction of eosinophils with apoptotic cells induced marked transcriptional changes in eosinophils that were associated with induction of an anti-inflammatory, immunosuppressive phenotype. These data are consistent with the overall understanding that engulfment of apoptotic cells induces potent immunosuppressive activities in immune cells. For example, the immunosuppressive activities of apoptotic cells upon their interaction with immune cells results in induction of various suppressive mechanisms including the secretion of potent anti-inflammatory cytokines [e.g., TGF-β and interleukin-10 (IL-10)] (50, 51). Similarly, apoptotic cells induced the expression of *Tgfb2* in eosinophils and decreased the expression of numerous pro-inflammatory chemokines/cytokines including *Cxcl1, Cxcl3 Cxcl9, Cxcl11, Ccl2, Ccl3, Ccl4 Ccl8, and Ccl22*, *Il18* and *Il27*. In contrast, chemokines/cytokines that are associated with anti-inflammatory responses including *Ccl17*, *Ccl24*, *Chil3*, and *Vegf* were increased. Furthermore, while the expression of several key proinflammatory transcription factors was decreased (e.g., *Stat1, Stat2, Nfkb1* and *Nfkb2*), the expression of inhibitory transcription factors [e.g., NFκB inhibitor alpha (*Nfkbia*)] and factors associated with tissue repair and wound healing were increased. It was previously shown that apoptotic cells upregulated the expression of *Nr4a1* in macrophages (52), which in turn inhibited the activation of NF-κB and Egr1 (52), which suppress pro-inflammatory gene expression. Notably, apoptotic cells upregulated the expression of *Nr4a1* and *Egr1* in eosinophils as well. Collectively, these data suggest that apoptotic cells do not induce a global “shut down” of eosinophil transcriptional activity but rather actively reprogram transcriptional activity that results in an anti-inflammatory immunosuppressive eosinophil phenotype.

Although the major cells that are responsible for efferocytosis and clearance of apoptotic cells are macrophages (10), eosinophils are posed in various anatomical locations such as the gastrointestinal tract and are present in multiple inflammatory conditions that are characterized by tissue damage and cell death including colitis, cancer, parasite infections and asthma (1, 2). We have recently shown that like macrophages, exposure of eosinophils to Type 1-associated cytokines (e.g., IFN-γ in the presence of bacterial stimuli) polarized them to display a pro-inflammatory phenotype (termed Type 1 eosinophils). In contrast, exposure to Type 2-associated cytokines (e.g., IL-4) drove them towards immunomodulatory activities (termed Type 2 eosinophils) (6). Whether apoptotic cells could further augment eosinophil polarization is unclear. Herein, we show that apoptotic cells markedly suppressed IFN-γ, *E. coli* and combination of IFN-γ+*E.* coli-induced inflammatory responses and secretion of inflammatory cytokines in eosinophils. In contrast, interaction of eosinophils with apoptotic cells augments their Type 2-associated signature, which is associated with tissue repair and wound healing. Our data are consistent with earlier studies using monocytes, which showed that co-culture of LPS-stimulated monocytes with apoptotic cells resulted in inhibition of TNF-α secretion and induction of IL-10 release (53). Furthermore, they are also in line with the recent finding that IL-4 or IL-13 alone were insufficient to induce an optimal tissue repair transcriptional program in macrophages. Rather, IL-4/IL-13 required the presence of apoptotic cells (14). Interestingly, exposure of eosinophils to IL-4 in the presence of apoptotic cells resulted in transcriptional profile that was different than the one observed for macrophages, which were stimulated with IL-4 in the presence of apoptotic cells and resulted in an overlap of only 6 transcripts (*Chil3*, *Retnla*, *Clec4a2*, *Rnase2a*, *Ddx4*, and *Ear2*). This was unexpected since the transcriptional profile of Type 2 polarized eosinophils was shown to display marked overlap and similarity with that of M2-polarized macrophages (6). The different transcriptional phenotype of eosinophils and macrophages in response to IL-4 and apoptotic cells likely results from the fact that macrophages express a large and different repertoire of receptors and intracellular molecules that can bind, engulf and digest apoptotic cells (54).

In addition to the 6 transcripts, which were shared with macrophages, additional transcripts that are associated with M2 macrophage activities were identified in our analysis. DAB adaptor protein 2 (*Dab2*) which has been shown to display immunosuppressive activities in macrophages and is involved in TGF-β signaling (55), was upregulated in eosinophils following activation of IL-4 in the presence of apoptotic cells. The expression of *Hivep3*, a transcription factor that negatively regulates gene expression and is suggested as a suppressor of pro-inflammatory gene expression in M2 macrophages (56), was augmented in response to IL-4 and apoptotic cells. Furthermore, expression of *Klf4*, a transcription factor that is associated with the suppression of M1 macrophage polarization and the promotion of M2 polarization (57), was upregulated in IL-4-activated eosinophils in the presence of apoptotic cells. Finally, several IL-4-induced surface molecules were augmented by apoptotic cells in eosinophils. Among these, we identified *Cd209* and *Car12*, which were previously shown to be expressed by M2 macrophags (58), and the alternative (IL-4)-activated macrophages marker, *Mrc1* (CD206) (58). Together, these findings indicate that IL-4 and apoptotic cells induce a distinct transcript signature in eosinophils that augments IL-4 induced responses and polarizes eosinophils into a cell that is associated with Type 2 immune responses such as tissue repair and remodeling.

Our study bears several limitations. First, we could not identify the cell surface receptor in eosinophils, which interacts with apoptotic cells. Nonetheless, we demonstrate that CD300b and CD300f are not involved in suppression of Type 1 eosinophil responses and augmentation of Type 2 eosinophil responses by apoptotic cells. One of our study limitations is that we cannot exclude possible involvement for CD300a especially since *Cd300a^-/-^* eosinophils did not response to *E. coli* stimulation. We did not use neutralizing antibodies to overcome this limitation since we are unaware of neutralizing antibodies capable of locking CD300a (or other CD300 family members). While the inability of CD300a to response to *E. coli* stimulation was unanticipated it was not surprising since we have previously shown that CD300 family members are required for eosinophil activation by IL-4 and IL-33 (20, 59). An addition limitation is that our efferocytosis assays were conducted for long time periods (~18 hrs). This time frame was specifically chosen to assess global transcriptome changes in eosinophils. Nonetheless, it may introduce the activation of various pathways in eosinophils that may be triggered by danger-associated molecular patterns (DAMPs) that in turn can activate a plethora of innate immune receptors. Finally, our study was conducted on murine eosinophils that have been obtained from the peritoneal cavity of *Il5Tg* mice or generated *in vitro* from BM progenitor cells. Thus, the relevance of these findings to human eosinophils remains to be determined. It will be of specific interest to determine whether eosinophils can engulf apoptotic cells in colon cancer where eosinophils have been suggested to directly kill tumor cells (16). Despite these limitations, our global sequencing approach, microscopy studies and efferocytosis assays demonstrate that eosinophils can bind, engulf, and functionally respond to apoptotic cells. Future studies should comprehensively characterize the molecular mechanisms, which enable the interaction of eosinophils with apoptotic cells.

Taken together, in this study we extend our knowledge regarding the transcriptional plasticity of eosinophils. We provide important insights into the regulation of eosinophil gene expression in distinct inflammatory environments and demonstrate the key regulatory effects of apoptotic cells on eosinophil activation especially in Type-1 and Type-2-associated immune settings.

## Data availability statement

The data presented in the study are deposited in the NCBI repository, accession numbers GSE189213 and GSE216110.

## Ethics statement

The animal study was reviewed and approved by Animal Health Care Committee of the Tel Aviv University and were performed in accordance with the regulations and guidelines regarding the care and use of animals for experimental procedures.

## Author contributions

DA and AM- conception and/or design of the work. AD, IH, SA, MI- Data Collection. AD, IH, SA, MI, AM- Data analysis and interpretation. AZ, FL-S- Critical reagents for the study. AD, AM- Drafting the article. AD, FL-S, AM - Revision of the article. AM- Final approval of the version to be published.

## Funding

This work was supported by grants and fellowships to MA from the US-Israel Bi-national Science Foundation (grant no. 2015163), Israel Science Foundation (grants no. 886/15 and 542/20), Israel Cancer Research Fund, Richard Eimert Research Fund on Solid Tumors, Israel Cancer Association, Dotan Hemato Oncology Fund, Cancer Biology Research Center, Tel Aviv University, The Tel Aviv University Faculty of Medicine Recanati Fund, and Azrieli Foundation Canada-Israel, and in part by grants to Francesca Levi-Schaffer from the US-Israel Bi-national Science Foundation (grant no 2015045.) and Aimwell Charitable Trust (UK).

## Conflict of interest

AM is a consultant for Glaxo Smith Kline, Astra Zeneca, Sanofi, Oravax, Sartorious and is an inventor of three patents owned by the Tel Aviv University.

The remaining authors declare that the research was conducted in the absence of any commercial or financial relationships that could be construed as a potential conflict of interest.

The handling editor YT declared a shared parent affiliation with the authors IZ and F-LS at the time of review.

## Publisher’s note

All claims expressed in this article are solely those of the authors and do not necessarily represent those of their affiliated organizations, or those of the publisher, the editors and the reviewers. Any product that may be evaluated in this article, or claim that may be made by its manufacturer, is not guaranteed or endorsed by the publisher.
